# The Medical Curriculum

**Published:** 1916-09-02

**Authors:** 


### THE MEDICAL CURRICULUM.

### QUALIFICATION AND REGISTRATION.

THE first thing a medical student should do is to register himself as such. Five years of study are necessary in order to obtain a qualification. Application for registration must be made to the Registrar of the General Medical Council, 44 Hallam Street, Portland Place, London, W.

The student, however, cannot be registered until he has (1) passed a preliminary examination in Arts which is recognised by the General Medical Council, and (2) has commenced medical study.

A complete list of the recognised preliminary examinations may be obtained on application to the Registrar of the General Medical Council (price 6½d.), and it is essential in any case to discover at a sufficiently early period what preliminary examinations are recognised by the particular examining body that it is proposed to work under. For example, with a few exceptions, the London Matriculation is the only preliminary examination which will permit a candidate to proceed to the necessary examinations in order to become a graduate of the University of London.

Wherever he proposes to study, and whatever qualification he intends to get, the courses of study which a medical student has to undergo are really very much the same; they may be divided, generally speaking, into the two groups of the preliminary and the clinical. But it is not, as a rule, compulsory to undergo the necessary courses of instruction in these two divisions of medical education at the same school. Often a student will be well advised to undergo his preliminary training at a provincial school, or as a student of the University of London, and to pursue his clinical studies elsewhere, as in one of the large Metropolitan hospitals.

The following is a list of the examining bodies, and the degrees and diplomas granted by them which admit the graduate or diplomate to enter his name in the register of legally qualified medical men :—

ENGLAND.

| EXAMINING BODY. | QUALIFICATION. |
|---|---|
| University of Oxford ... | M.B., B.Ch. (only obtainable after graduating in Arts). |
| University of Cambridge ... | M.B., B.C. |
| University of Durham ... | M.B., B.S. |
| University of London ... | M.B., B.S. |
| Victoria University of Manchester | M.B., Ch.B. |
| University of Birmingham... | M.B., Ch.B. |
| University of Liverpool ... | M.B., Ch.B. |
| University of Leeds ... | M.B., Ch.B. |
| University of Sheffield ... | M.B., Ch.B. |
| University of Bristol ... | M.B., Ch.B. |
| Conjoint Examining Board in England | M.R.C.S., L.R.C.P. |
| Society of Apothecaries of London | L.M.S.S.A. |

SCOTLAND.

| | |
|---|---|
| University of Edinburgh ... | M.B., Ch.B. |
| University of Glasgow ... | M.B., Ch.B. |
| University of Aberdeen ... | M.B., Ch.B. |
| University of St. Andrews... | M.B., Ch.B. |
| Conjoint Examining Board in Scotland | L.R.C.P.Edin., L.R.C.S. Edin., and L.R.F.P.S. Glasg. |

IRELAND.

| | |
|---|---|
| University of Dublin ... | L. Med., Lic. Surg. (conferred on those who have not graduated in Arts), M.B., B.Ch. (only conferred on graduates in Arts). |
| National University of Ireland | M.B., B.Ch. |
| Queen's University, Belfast | M.B., B.Ch. |
| Conjoint Examining Board in Ireland | L., L.M., R.C.P.I., L., L.M., R.C.S.I. |
| Apothecaries' Hall of Ireland | L.A.H.Dub. |

WALES.

| | |
|---|---|
| University of Wales ... | M.B., B.Ch. |

The B.A.O. degree, though granted by certain Universities, is not legally registrable.

### THE ENGLISH CONJOINT COURSE.

THE student who is desirous of taking the Conjoint qualification—that is to say, the double diploma of Member of the Royal College of Surgeons of England and Licentiate of the Royal College of Physicians of London—has to comply with the regulations laid down for medical qualifications generally. When registered as a medical student he has to show that he has attended lectures and practical work in chemistry, physics, and biology before he is allowed to proceed to the first, second, or third part of his "first." If he wishes to take the fourth part at the same time he has to show proof that he has been properly instructed in practical pharmacy by a recognised chemist or registered practitioner. The subjects for the first examination are chemistry, physics, biology, and pharmacy. In the first two parts the examination is partly written and partly practical; in the third part it is written and oral, and in the fourth part it is wholly oral. The practical chemistry consists of simple quantitative and qualitative analysis, and candidates usually find the test a comparatively easy one. Assuming that the student has had some preliminary training, such as can usually be obtained at the general schools from which he passes his preliminary, he ought not to take a longer time than six months to pass the first three parts of the "first." Many students take these parts after only three months' work. The biology required for the first examination is exceedingly elementary.

Having passed the first two parts of it, the student is allowed to enter the dissecting-room and commence his study of anatomy and physiology. He must be engaged in the study of these two sciences for twelve months during the ordinary session before he can enter for his second examination, and during that time he must have actually dissected the several "parts," and must have attended a course of instruction in histology and practical physiology, together with a course of lectures (six months) in physiology and anatomy at a recognised medical school.

The second examination is both oral and written. There are two papers, one in each subject, and each has six questions, of which the candidate must answer at least four within the stipulated three hours. Here, too, as in the "first," the questions are straightforward, and the student who has displayed average diligence in his studies should not have much difficulty in satisfying the examiners at the end of his second year. The *viva voces* are purely objective, and the candidate is rarely asked to describe any structure.

For the final or qualifying examination the student must produce evidence of having attended at a recognised medical school specified courses of lectures on medicine, surgery, midwifery, pathology (including pathological histology and bacteriology), pharmacology and therapeutics, forensic medicine and insanity, and public health; of having received systematic practical instruction in these subjects; and of having performed operations on the dead body. In addition he must show that he has attended, since passing his second examination, the practice of medicine and surgery, demonstrations in the post-mortem room, clinical lectures on medicine and surgery and diseases of women, and of having served as clerk and dresser in the wards. He must also have obtained a certificate of instruction and proficiency in the administration of anæsthetics, and have attended special classes in ophthalmology, in fevers, in lunacy, and in vaccination, and must have conducted twenty labours. In addition he must be twenty-one years of age or older.

The examination is in three parts, medicine, surgery, and midwifery, and the student is allowed to take the examination at the completion of five years of medical study. The examination comprises four papers of six questions, and a *viva voce*. In surgery and medicine there are additional *viva voces* in anatomy and pathology, together with a long *viva* on "clinical cases."

### THE FELLOWSHIP OF THE ROYAL COLLEGE OF SURGEONS.

The Fellowship of the Royal College of Surgeons of England is justly esteemed as the premier qualification that the student can obtain in surgery, and is almost an essential to anyone who aspires to the higher surgical posts in England. The student should decide early in his career whether he wishes to obtain the F.R.C.S. For this purpose it is necessary to pass two examinations, a primary and a final.

The Primary examination takes place twice every year, in May and in November, and is held in London. The subjects are anatomy and physiology, and candidates must have passed the second Conjoint examination or some other equivalent examination which exempts them from it. They must also (except in the case of members of the college) have spent three winter sessions or eighteen months in dissections and attendance at physiology classes at a recognised school. The fee for the examination is £5 5s. The examination is partly oral and partly written. Four papers are set, two in physiology and two in anatomy, four questions, of which only two must be answered, being given with an allowance of two hours for each paper. The oral examinations are two, lasting twenty minutes each, in the two subjects. The examination has the reputation of being unusually stiff, but its difficulty should not frighten the student or practitioner of reasonable ability who is willing to pay special attention to the two subjects. A good, sound knowledge of anatomy and physiology is required, and if the candidate has been diligent at his practical work in the dissecting-room and laboratory, and has supplemented the knowledge there gained by reading, he stands a good chance of success.

The examination for the Final Fellowship takes place in May and November of each year, and consists of a written paper, *viva voce*, clinical examinations, and operative surgery. The fee for the examination is £12 12s., and candidates who are members of the college are admissible when they have been six years in the study or in the study and practice of medicine. Other candidates must show that they are graduates in medicine of recognised Universities of four years' standing, and have attended the surgical practice of a recognised hospital for one year after graduation. Special classes for the Final Fellowship examination are held at most of the London hospitals; and practitioners who intend to enter for it, and have lost touch to any extent with hospital surgical practice, would do well to attend a full course of instruction at one of the medical schools before competing.

### LONDON UNIVERSITY COURSE.

The University confers the degrees of Bachelor of Medicine and Bachelor of Surgery (M.B., B.S.), Doctor of Medicine (M.D.), and Master of Surgery (M.S.).

The student who desires to take the degree of M.B. of the London University has to pass the University Matriculation Examination, held thrice yearly in January, September, and June. The matriculation is accepted as a preliminary examination for graduation or qualification in medicine, provided the candidate has taken Latin as one of the optional subjects, and formerly no other examination exempted from the matriculation so far as degrees of the University were concerned. Recently, however, the Senate has seen fit to rule that several other examinations may be accepted instead of the usual matriculation.

After matriculating the student must attach himself to one or other of the various medical schools, and devote himself during his first year to mastering the subjects—chemistry, biology, and physics—required for his preliminary scientific or first professional examination. It will take him a full year to work up these subjects.

The student is at present admitted to the Intermediate examination, the subjects of which consist of anatomy, physiology, and pharmacology, two years after having passed Part I. of the Preliminary. Students who have passed the B.Sc. (Pass) Examination with physiology, or who have obtained honours at the B.Sc. Examination, having taken the Pass Examination in physiology in respect of their subsidiary subject, will not be required to pass the physiological portion of the Intermediate Examination for M.B. Time spent in working at the subjects of anatomy and physiology is not recognised by the University until the student has passed the Preliminary. His second and third years are thus spent in work in the dissecting room and physiological laboratory.

Having passed his "Inter.," the student begins clinical work. Two years of clinical study were formerly required, but now three years will be necessary, while two and a half years will be the minimum period required for the preliminary subjects. Thus the full course will in future occupy at least five and a half years from the time of matriculation. As a special war measure the regulations have been relaxed in certain ways in favour of those presenting themselves for their Final Examinations. The full details of these relaxations of the regulations are contained in a blue pamphlet which can be obtained from the University authorities.

The last examination is written, oral, and practical. The candidate is required to examine and report on selected cases, to do practical bacteriological work, and to be acquainted with the essentials of operative surgery, though actual operations on the dead subject are not demanded.

### OXFORD UNIVERSITY COURSE.

At Oxford University two degrees in medicine can be obtained, the B.M. and the D.M.; similarly in surgery there are two degrees, the B.Ch. and the M.Ch. No candidate is eligible for any of these degrees unless he is a graduate in Arts, that is to say, either an M.A. or a B.A.; in which branch of the Arts that degree has been obtained is a matter of little moment. The usual course of the medical student who has passed Responsions is to work at the Preliminary Science examinations in physics, chemistry, and zoology and botany, which are likely to take him two years or thereabouts, and then to spend a further two years in study for the Final Honour School of physiology, in which he takes his degree of B.A. He next has to pass examinations in organic chemistry in relation to medicine and in human anatomy, after which he prepares himself for his second professional examination, which comprises the subjects of materia medica and pharmacology, pathology, forensic medicine and hygiene, and the Final subjects of medicine, surgery, and midwifery. When the examinations in these subjects have been passed, the candidate may supplicate for and obtain the degree of B.M., B.Ch.

The degree of D.M. is granted to Bachelors of Medicine of the University who have entered their thirty-ninth term —it should be remembered that there are four terms in the official year—who have presented a dissertation on some medical subject that is approved by the Professors of the Faculty and Examiners for the degree of Bachelor of Medicine for the time being whose subjects are dealt with in it. The degree of M.Ch. is granted to Bachelors of Surgery of the University who have entered their twenty-seventh term and satisfied some other requirements; the examination for this degree is held annually in June.

### CAMBRIDGE UNIVERSITY COURSE.

The degrees of Bachelor of Medicine (M.B.) and Bachelor of Surgery (B.C.) at this University are only conferred after the entire course of study with examinations for both has been carried out. The B.C. is, therefore, in itself a complete and registrable qualification to practise, and is frequently so used. To obtain either of these degrees it is necessary to pass five years in the study of medicine after registration as a student, and to reside for three years at the University. The professional examinations are three in number. The first comprises physics, chemistry, and biology; like similar examinations elsewhere, it is divided into three parts, which may be passed separately. The second is divided into two parts—Part I. comprising human anatomy and physiology, Part II. elementary pharmacology and pharmaceutical chemistry and general pathology. The third is also divided into two parts—Part I. including surgery and midwifery, Part II. physic and pathology and pharmacology.

When the third examination has been completed the student may take the degree of B.C. at once, but for the M.B. he must prepare and submit a thesis on some subject in medicine, surgery, or midwifery, selected by himself.

For the degree of M.D. it is necessary to have been M.B. at least three years, and to submit a thesis on some subject in medicine (not in surgery) which is a definite research or contribution to the knowledge of its subject.

There is also an advanced degree in surgery (Master of Surgery, M.C.), which is said to demand a very distinctly higher standard of surgical knowledge and proficiency than the English F.R.C.S. Not many candidates attempt it, and fewer still attain it; practically all who possess it are surgeons on the staff of important general hospitals.

### HIGHER DIPLOMAS.

**Royal College of Physicians of London** grants its membership after examination to graduates in medicine of recognised Universities, or to licentiates of the College, being above the age of twenty-five years, who do not engage in trade, do not dispense medicine, and who do not practise in partnership. The examination, which is held in January, April, July, and October, is partly written and partly oral. The fee for the membership is £42, but if the candidate is a licentiate the fee is the difference between what he has already paid and £42. In either case £6 6s. is paid before examination. The fellowship is elective.

**Royal College of Physicians of Edinburgh** admits to its membership licentiates of the College, or of a British or Irish College of Physicians, or graduates in medicine of a British or Irish University over twenty-four years of age after examination in medicine and therapeutics and one or more of the following subjects selected by the candidate: (1) One or more departments of medicine specially professed, (2) psychological medicine, (3) general pathology and morbid anatomy, (4) medical jurisprudence, (5) public health, (6) midwifery, (7) diseases of women, (8) diseases of children, (9) tropical medicine. The fee to be paid by a licentiate is twenty guineas, by others thirty-five guineas. A member of three years' standing may be elected a Fellow. The fee is £64 18s.

**Royal College of Surgeons of Edinburgh.** The Fellowship is granted, after examination, to any registered practitioner who is twenty-five years of age, and has been in practice for two years, on petition being granted. The examinations are written, oral, and practical. The fee is £35 to those who hold the diploma of licentiate of the College, and £45 to others (no stamp duty is payable on the diploma). Apply for particulars to Mr. D. L. Eadie, 50 George Square, Edinburgh.

**Royal Faculty of Physicians and Surgeons of Glasgow.**—Registered practitioners are admitted to the Fellowship by examination and by subsequent election. Four examinations are held annually (January, April, July, October). Fourteen days' notice must be given. The fee is £30 unless the candidate desires to qualify to hold office, when it is £50. In the case of a licentiate of the faculty, £25 and £15 respectively.

**Roya College of Surgeons in Ireland Fellowship.**—(1) The examination for the Fellowship is divided into two parts—namely, the primary and the final. (2) The subjects of the Primary examination are anatomy (including dissections), physiology, and histology. The examination is partly written, partly *viva voce*, and partly practical. Candidates must pass in all the subjects at one examination. (3) The subjects of the Final examination are surgery (including surgical anatomy) and pathology. The examination is partly written and partly *viva voce*, and includes the examination of patients and the performance of operations on the dead body. Candidates must pass in all the subjects at one examination. (4) The examinations are held three times in each year. (5) Special examinations will not be granted by the Council under any circumstances. (6) Candidates are required to lodge their applications, certificates, and receipts for fees with the Registrar at least seven days before the date of the examination.

